# Host variations in SARS-CoV-2 infection

**DOI:** 10.3906/biy-2104-67

**Published:** 2021-08-30

**Authors:** Doruk ALTIOK, Elif Zeynep SAVCI, Büşra ÖZKARA, Kamil ALKAN, Dilara Sultan NAMDAR, Gizem TUNÇER, Buğrahan Regaip KILINÇ, Evren SUİÇMEZ, Güneysu ÇETİN, Sinan ÜNAL, Beyza DÖNMÜŞ, Zeynep Yağmur KARAGÜLLEOĞLU, Dilruba Beyza UNCUOĞLU, Cansu TEKELİ, Hanife Ayşegül MENDİ, Vahdi Umut BENGİ, Güldane CENGİZ SEVAL, Pelin KILIÇ, Evrim GÜNEŞ ALTUNTAŞ, Devrim DEMİR-DORA

**Affiliations:** 1 Faculty of Dentistry, Başkent University, Ankara Turkey; 2 Faculty of Medicine, Akdeniz University, Antalya Turkey; 3 Faculty of Medicine, Süleyman Demirel University, Isparta Turkey; 4 General Biology Program, Graduate School of Science and Engineering, Hacettepe University, Ankara Turkey; 5 HücreCELL Biotechnology Development and Commerce, Inc., Ankara Turkey; 6 Department of Genetics and Bioengineering, School of Engineering and Architecture, Kastamonu University, Kastamonu Turkey; 7 Department of Biomedical Engineering, School of Engineering and Architecture, Kastamonu University, Kastamonu Turkey; 8 Department of Molecular Biology and Genetics, Graduate School of Science and Engineering, Yıldız Technical University, İstanbul Turkey; 9 Department of Molecular Biology and Genetics, Faculty of Science, Bilkent University, Ankara Turkey; 10 Department of Molecular Biology and Genetics, Faculty of Arts and Science, Yıldız Technical University, İstanbul Turkey; 11 Department of Biology, Graduate School of Natural and Applied Sciences, Ankara University, Ankara Turkey; 12 Department of Biomedical Engineering, School of Engineering, TOBB University of Economics and Technology, Ankara Turkey; 13 Department of Basic Sciences, Division of Medical Microbiology, Faculty of Dentistry, Gazi University, Ankara Turkey; 14 Department of Periodontology, Faculty of Dentistry, Gülhane Training and Research Hospital, Ankara Turkey; 15 School of Medicine Department of Hematology, Ankara University, Ankara Turkey; 16 Stem Cell Institute, Ankara University, Ankara Turkey; 17 Biotechnology Institute, Ankara University, Ankara Turkey; 18 Department of Medical Pharmacology, Faculty of Medicine, Akdeniz University, Antalya Turkey; 19 Department of Gene and Cell Therapy, Health Sciences Institute, Akdeniz University, Antalya Turkey; 20 Department of Medical Biotechnology, Health Sciences Institute, Akdeniz University, Antalya Turkey

**Keywords:** Angiotensin-converting enzyme 2 (ACE2), host genetics, polymorphisms, SARS-CoV-2, toll-like receptors (TLR), transmembrane serine protease 2 (TMPRSS2)

## Abstract

The novel severe acute respiratory syndrome coronavirus 2 (SARS-CoV-2), the zoonotic pathogen that causes the “Coronavirus Disease of 2019 (COVID-19)”, and COVID-19 itself is yet to be thoroughly understood. Both the disease as well as the mechanisms by which the host interacts with the SARS-CoV-2 have not been fully enlightened. The epidemiological factors –e.g. age, sex, race-, the polymorphisms of the host proteins, the blood types and individual differences have all been in discussions about affecting the progression and the course of COVID-19 both individually and collectively, as their effects are mostly interwoven. We focused mainly on the effect of polymorphic variants of the host proteins that have been shown to take part in and/or affect the pathogenesis of COVID-19. Additionally, how the procedures of diagnosing and treating COVID-19 are affected by these variants and what possible changes can be implemented are the other questions, which are sought to be answered.

## 1. Introduction

The novel severe acute respiratory syndrome coronavirus 2 (SARS-CoV-2), the zoonotic pathogen that causes the “Coronavirus Disease of 2019 (COVID-19)”, and COVID-19 itself is yet to be thoroughly understood. Both the disease as well as the mechanisms by which the host interacts with the SARS-CoV-2 infection have not been fully enlightened. 

An important feature of the host is its genetic structure. Although genetic is a vast area of biological sciences, one object of interest can be identified regarding the medical implications of this field: polymorphisms. Polymorphism, which is described as the occurrence of a variation in the genomic sequence where the infrequent variant is present in 1% of the people being tested^1^NHGRI (2020). Talking Glossary of Genetic Terms, Polymorphism [online]. Website: www.genome.gov/genetics-glossary/Polymorphism [accessed 4 November 2020]., is one of the centerpieces of genetic research. There is an innumerable amount of research regarding this topic, e.g. the studies on the effects of protein polymorphisms on the susceptibility to SARS. To expand this exemplary subject of SARS research, polymorphic variants of host proteins such as dendritic cell-specific intercellular adhesion molecule-3-grabbing non-integrin (DC-SIGN) (Chan et al., 2010), intracellular adhesion molecule 3 (ICAM-3) (Ka et al., 2009), angiotensin converting enzyme 2 (ACE2) (Chiu et al., 2004) and tumor necrosis factor α (TNF-α) (S. Wang et al., 2008), and their roles in the SARS infections were heavily studied after the SARS-CoV outbreak in 2002. Along the lines of these articles, several studies regarding the polymorphisms of the viral genome were also conducted. For example, Shang et al. searched for polymorphisms in 116 genomes of SARS-CoV and compared the BJ202 and GZ02 genomes (Shang et al., 2006). Considering the fact that SARS-CoV and SARS-CoV-2 share 79.5% of each other’s DNA (Suryamohan et al., 2021), one can question the role of host and virus genetics in the SARS-CoV-2 infection and COVID-19, just the same as the questions that were raised in the times of the SARS outbreak.

Similar to SARS and the Middle Eastern Respiratory Syndrome (MERS), COVID-19 presents with dry cough, fever, ground glass opacities on chest computerized tomography (CT) scans and dyspnea (Huang et al., 2020). Another similarity is that SARS-CoV-2 and its namesake SARS-CoV both use the ACE2 protein for entering the host cells (Ge et al., 2013; Hoffmann, Kleine-Weber, Krueger et al., 2020). Since these symptoms and the viruses’ cell entry mechanisms are associated with the respiratory system, environmental and personal factors, e.g., smoking, diet ary habits, which affect this system, are other areas of COVID-19 that need to be clarified. However, in studies focusing on the genetic affinity between SARS-CoV and SARS-CoV-2, it should be noted that clinical data differ between SARS-CoV and SARS-CoV-2 due to the fact that there are variations between the rates of infection, virulence, and transmission of these two viruses (Rossi et al., 2020). In addition, serious data sources were created for SARS-CoV-2, various biological databases are currently in use to search both proteins and the genome of SARS-CoV-2, which holds geographical and phylogenetic data of variants and mutants of this virus. Since there are a lot of data for SARS-CoV-2 compared to SARS-CoV, the differences between the genetic analysis approaches for SARS-CoV and SARS-CoV-2 should be taken into account when evaluating polymorphic variants. For instance, https://grasp.nhlbi.nih.gov/Covid19GWASResults.aspx provides many genomewide association analysis (GWAS) data for immediate use in COVID-19. Even if the association of the infection with respiratory failure is poorly understood, higher mortality is widely linked to older age and the male sex (Li et al., 2020; F. Zhou et al., 2020). Hypertension, diabetes, and other obesity-related and cardiovascular disease traits have also been found in association with the infection. However, the relative role of clinical risk factors in determining the severity of COVID-19 has not yet been clarified. Hence, GWAS association analysis (GWAS) may be a crucial tool in identifying genetic factors involved in the development of the infection (R. Chen et al., 2020; Docherty et al., 2020; “Genomewide Association Study of Severe Covid-19 with Respiratory Failure,” 2020; Q. Li et al., 2020; S et al., 2020; F. Zhou et al., 2020).

We asked the abovementioned questions with the aim to answer them in the occasion of this study. We focused mainly on the effect of polymorphic variants of the host proteins that have been shown to take part in and/or affect the pathogenesis of COVID-19. Additionally, how the procedures of diagnosing and treating COVID-19 are affected by these variants and what possible changes can be implemented are the other questions, which are sought to be answered.

## 2. Angiotensin converting enzyme 2 (ACE2)

ACE2 was first identified as the angiotensin converting enzyme (ACE) homologous, which has zinc metalloproteinase activity. It is now known that it has numerous activities different from those of the ACE (Burrell et al., 2012). It is a major regulator in cardiac function (Burrell et al., 2012) and it was also discovered to be a functional receptor of SARS-CoV (Ge et al., 2013).

While the spike (S) protein of MERS-CoV uses dipeptidyl peptidase-4 (DPP4) as the attachment molecule, S protein of SARS-CoV and SARS-CoV-2 tend to use the human ACE2 as their main receptor (F. Li, 2015; R. Yan et al., 2020). This means that SARS-CoV-2 uses the ACE2 membrane protein to enter the cell with a high efficiency and infect the organism (Corley & Ndhlovu, 2020; Wan et al., 2020). It is stated that SARS-CoV-2 S proteins show more affinity towards ACE2 than SARS-CoV does, and this may have made SARS-CoV-2 more contagious than SARS-CoV (Chakraborty, 2021; Delgado et al., 2021; Suryamohan et al., 2021; Wrapp et al., 2020). However, this statement opposes the conclusion that was drawn by other researchers, which argue that the affinities of SARS-CoV-2 and SARS-CoV to ACE2 are similar (Lan et al., 2020; Walls et al., 2020). In addition, twenty natural ACE2 variants identified can positively affect the susceptibility of the virus by changing the interaction between the virus and the host, and play an important role in this situation (Suryamohan et al., 2021). In addition, 20 natural ACE2 variants identified can positively affect the susceptibility of the virus by changing the interaction between the virus and the host and play an important role here (Suryamohan et al., 2021).

Overall, the ACE2 population variants that increase or decrease sensitivity have been reported to be rare, and this rarity has been reported to be consistent with the overall low appearance of the ACE2 receptor polymorphisms. But it is paramount to associate the ACE2 genotypes and the clinical pictures in a population-wide manner in order to understand the importance of the ACE2 variations in susceptibility for such infections (Suryamohan et al., 2021).

### 2.1. ACE2 variants and spike proteins

When the interaction between the human ACE2 variants and the SARS-CoV-2 S proteins are investigated, using homology modeling and superimposition methods, which focused on whether the ACE2 variants have a potential effect on the susceptibility to the infection, 3 alleles of 17 variants that were included in recent research were found to affect the attachment stability. The variants attached almost the same way, topologically. This is not surprising when it is considered that there are no significant differences between the backbones of these variants (Hussain et al., 2020). No pathogenic results were found regarding to these alleles (Hussain et al., 2020). Via the superimposition method, different variants were observed on the 6LZG complex (Johansson et al., 2012). 

The binding affinity is higher between SARS-CoV-2 and ACE2 compared to that between SARS-CoV and ACE2 (Gheblawi et al.,2020). The binding between ACE2 and the S protein of the SARS-CoV-2 are instinctively high to stabilize this connection at the whole-virus level (Yang et al.,2020). In addition, mutations of SARS-CoV-2 also have differences in binding affinity to ACE2, for example, the N501Y mutant is known to have greater binding affinity compared to other mutant types (Ali et al., 2021). This difference is not only between SARS-CoV-2 mutants and human ACE2, but also at the interaction of SARS-CoV-2 with ACE2 in terms of other organisms: the binding capacity of the human ACE2 proteins with SARS-CoV-2 is relative to mammals that are genetically close - such as mice and rats- is higher (Indu et al., 2020). The difference between the mutant types’ affinities for binding to ACE2 causes the virus variants, including mutations, to interact with the ACE2 protein at a different rate, for instance, the B.1.1.7 and B.1.351 variants are more advantageous compared to other variants in terms of binding affinity with ACE2 (Ramanathan et al., 2021). Compared to the viruses that are closely related to SARS-CoV-2, such as NL63, SARS-CoV, the advantage of the ACE2 binding of SARS-CoV-2 can be explained by reasons such as hydrophobic cluster formation and importance of binding site residues (Rawat et al.,2021). Considering this situation of high binding affinity between SARS-CoV-2 and ACE2 in the receptor-binding domain (RBD) axis, although the SARS-CoV RBD is in a more compact form compared to the SARS-CoV-2, some residual changes in the SARS-CoV-2 RBD seem to affect the interaction of the SARS-CoV-2 RBD and ACE2, which is known to stabilize the SARS-CoV-2 RBD and ACE2 interface (Shang et al., 2020b).

### 2.2. Non-Human ACE2 Polymorphisms 

**Figure 1:**
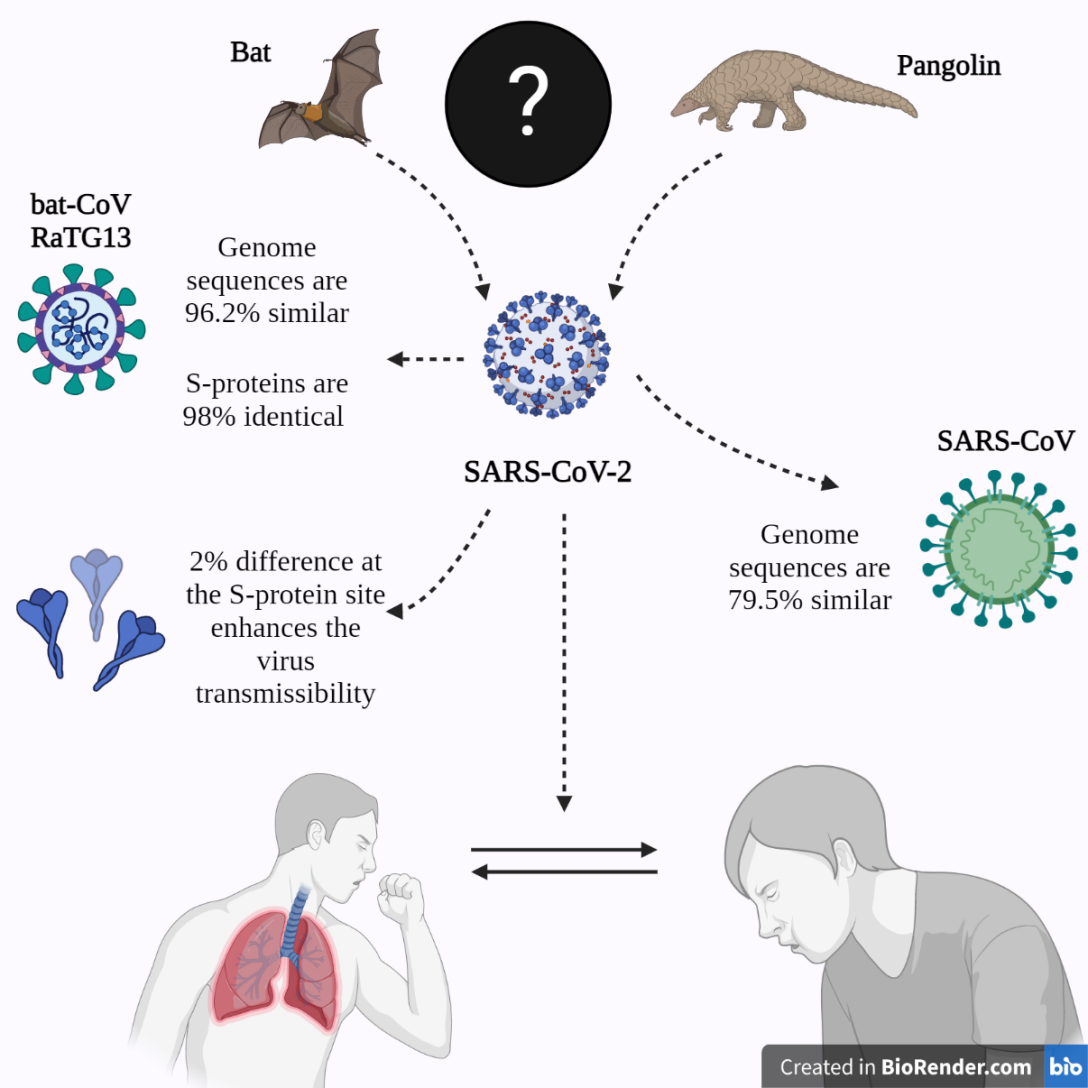
The possible origin of SARS-CoV-2 and its similarities: An illustration showing the genomic similarities shared by SARS-CoV-2, SARS-CoV and bat-CoV RaTg13. The illustration also displays the disputed origin of the SARS-CoV-2: the bat and/or the pangolin (Lam et al., 2020; Othman et al., 2020).

When we focus on the ACE2 polymorphisms in different species, according to the genome sequencing analysis results pursued by Suryamohan et al., (2021), the virus shares the closest similarity, up to 96.2%, with “bat-CoV RATG13” (Suryamohan et al., 2021). Similarities between SARS-CoV-2 and SARS-CoV are found to be around 79.5%. According to such data, an assumption can be made that the virus originates from bats, and, for a reason still unknown, it has been carried to humans with another intermediary (Suryamohan et al., 2021), possibly through a pangolin (Lam et al., 2020) (Figure 1). Some additional evidence is found, however, when the interaction of the different S proteins of the different coronaviruses and the different host ACE2s are observed via in silico studies; it is said that the receptor binding domain (RBD) of SARS-CoV-2 obtained its interface segment via the complex evolutionary processes rather than a mutation buildup. When several CoV S protein RBDs, including bat CoVs and other suspected intermediate host CoVs like the pangolin and palm civet, were investigated, crucial modifications in the SARS-CoV-2 infection of humans were observed (Xia et al., 2020; R. Yan et al., 2020). Besides, by examining eight different variants at the interaction surface of ACE2, it was found that none of these variants would impair the interaction with the viral RBD of SARS-CoV-2. In RBD multi-sequencing analyses, it was observed that the interface segment of SARS-CoV-2 shows more similarity with RatG13, SZ16, ZS-C, WIV16, 101 MA15, and SARS-CoV-Sino1-11 isolates rather than the Rm1, the Bat-SL-CoVZC45, and 98 Bat-SL-CoVZXC21 isolate (Othman et al., 2020). Antony and Vijayan (2021) have suggested that we may come across genetic variations in ACE2, which could affect the expression level as well as protein conformation and stability, which can also alter the SARS-CoV-2 S protein affinity. Accordingly, this may cause humans to be more resistant or susceptible (Antony & Vijayan, 2021).

### 2.3. Individual differences and their impact on ACE2

#### 2.3.1. Sex

There is a consistency with the finding that the conversion of Angiotensin (Ang) II to Ang 1-7 by ACE2 is higher in males than in females, suggesting an over-expression of ACE2 in males, although there are some studies claiming that there is no correlation between sex and ACE2 expression (Chen et al., 2020; Devaux et al., 2020; Gibson et al., 2020). Zhao et al., (2020), mentioned that the ACE2 expression profiles in normal lung cells in Asian males have a higher expression of ACE2 compared to the Caucasian and African populations (Zhao et al., 2020).

**Figure 2:**
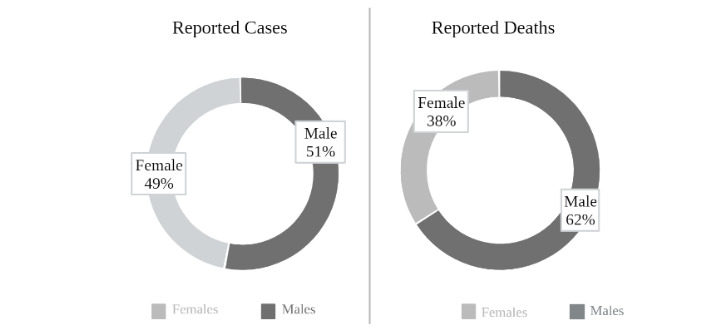
Epidemiological status in Turkey (patients/deaths). Pie charts showing the sex distribution of the reported cases and reported deaths in Turkey. Epidemiological data are taken from the “COVID-19 Weekly Situation Report (19 October 2020 – 25 October 2020)” published by the Ministry of Health of Turkey.^2^Republic of Turkey Ministry of Health (2020). COVID-19 Weekly Situation Report 19/10/2020 – 25/10/2020 Turkey [online]. Website: https://dosyamerkez.saglik.gov.tr/Eklenti/39230,covid-19-weekly-situation-report---43pdf.pdf?0&_tag1=D3D202441F1F5165A33D16981E6544EF7FC0A32F [accessed 4 November 2020]. These distributions may help to assess both the sex and ethnicity-based differences in prognosis.

There are scientists who have come to the conclusion that if the sex effect on mortality was considered, some connections may be deemed possible between the higher death ratio in males caused by SARS and MERS and the ACE2 X-linked inheritance (Darbani, 2020; W. Gibson et al., 2020; Q. Li et al., 2020). Additionally, pregnancy, sex chromosomes, estrogen signalling, and the ACE2 receptor levels are also proposed to be advantageous factors in females when compared to male (Stelzig et al., 2020). The fact that the human ACE2 protein is encoded on the X chromosome means that the males who carry rare ACE2 variants will express those variants in all ACE2-expressing cells, while females will typically express those variants in a mosaic distribution determined by early X-inactivation events (Gibson et al., 2020). Since ACE2 is X-linked, the rare variants that enhance SARS-CoV-2 binding in vivo would likely increase susceptibility to COVID-19 in males (Gibson et al., 2020). The X chromosome inactivation of the female body causes a “mosaic pattern”, meaning that half of the cells have the paternal X chromosome inactivated, and the other half has the maternal chromosome inactivated. This mosaicism may be in the advantage of the female host in terms of local inflammation and viral interaction (Gemmati et al., 2020). Other functional effects are also possible; as an example, the female variant carriers might have a shorter asymptomatic duration, or be more likely to develop symptoms, regardless of the viral shedding status (Gibson et al., 2020). Moreover, due to the X-linked phenotype, interaction booster and inhibitor ACE2 variants, which have been discovered by Darbani (2020), tend to being expressed more often in males rather than in females (Darbani, 2020). The effect of ACE2 becomes stronger via protease enzymes (cathepsin B (CTSB), cathepsin L (CTSL), type II transmembrane serine protease (TMPRSS2) that are determined by autosomal heredity because there is no difference between the sex dependent expressions of ACE2 variants in asexual tissues (Darbani, 2020). However, a study done on male and female rats showed that there is a sex difference in basal ACE2 activity selectively in the kidneys. The reported difference states that ACE2 in female kidneys are lower due to the hormonal effects of the ovaries mainly related to estradiol (Liu et al., 2010). These contradicting statements underlie the fact that this question of sex-based susceptibility via ACE2 needs to be thoroughly investigated. Ultimately, SARS-CoV-2 can affect males and females equally; however, the prognosis might be worse in males (M. Y. Li et al., 2020; Q. Li et al., 2020) (Figure 2). 

#### 2.3.2. Ethnicity

The fact that China is the birthplace of both the SARS and COVID-19 pandemics has raised some questions regarding the idea that maybe Asians are somehow more susceptible to these coronaviruses. According to Devaux et al., (2020), Asian people have higher ACE2 levels than the Caucasian and African-American populations. Asian people have higher ACE2 levels than the Caucasian and African-American populations. (Devaux et al., 2020) However, it was discovered by Chen et al. (2020), that the Asians are not different from other populations in terms of ACE2 expressions (Ying Chen et al., 2020). The controversy, hence, continues.

Studies focusing on ethnicity were continued by Li et al., (2020), by investigating the Asian and Caucasian populations in respect to ACE2 polymorphisms. They examined whether there is a variety between ACE2 expressions in the peripheral blood of 8 different populations, to discover genetic polymorphisms and variations in expressions of ACE2 (Q. Li et al., 2020). They also observed the ACE2 variations in terms of expression levels. However, as the differences were not significant, it could not be understood whether susceptibility would be caused in different populations (M. Y. Li et al., 2020).

Moreover, Darbani (2020), pursued another research using data from 4 human populations, including Europeans, Asians, Africans and Americans (Darbani, 2020). Accordingly, there are population-specific and non population-specific variants of ACE2. Some of the discovered variants were acting as an interaction booster, and these variants showed a some variation between populations. Some of the variants showed inhibitory characteristics, and these also showed some of the variations across populations (Table 1). However, the effect of the Asian specific variants to viral infectivity are unclear (Darbani, 2020). Therefore, the Asian population is not included in Table 1.

**Table 1:** Population based ACE2 variant characteristics.

	Interaction Booster	Inhibitor
European	H378R	-
African	S19P	M82I
American	-	Q388L

#### 2.3.3. Age

ACE2 has a positive correlation with age on the middle aged and elderly population, meaning that the susceptibility increases with age (Ying Chen et al., 2020), although SARS-CoV-2 can infect young and old people, equally. It is also seen that when the ACE2 expression levels between younger and older populations (≤49 years and >49 years, respectively) are compared, there is no statistically significant difference between the groups in any tissue (M. Li et al., 2020). From another perspective, O’Driscoll et al., (2020), examined age-specific COVID-19-associated death data from 45 countries. They found that the age distribution of deaths in younger age groups less than 65 years is very consistent. However, when going to ages over 65 years, they observed heterogeneous infection fatality patterns across countries (O’Driscoll et al., 2020). 

#### 2.3.4. Tissue types and ACE2

**Figure 3:**
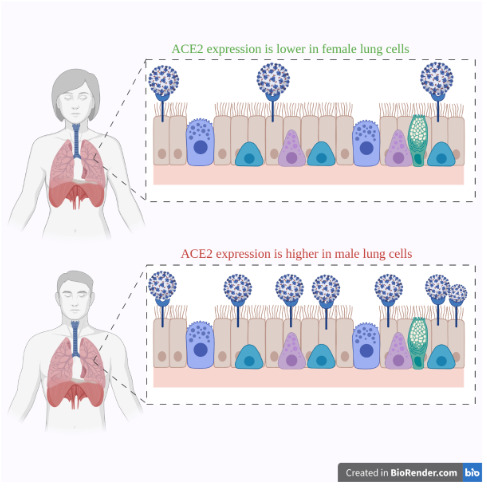
ACE2 expression in lung tissues.

ACE2 is found in cardiomyocytes, cardiac fibroblasts, pericytes, the vascular endothelium, and vascular smooth cells (Bavishi et al., 2020; Bojkova et al., 2020). When it comes to the ACE2 expression differences between tissues, in a research where CD8(+) cells, interferons (IFNs) and natural killer (NK) cells were used as indicators (M. Li et al., 2020), it was found that although the inflammation of the lungs is the primary symptom of patients with the SARS-CoV-2 infection, the lungs had moderate expression of ACE2 when compared with all the other tissues, and also it was noticed that males have a higher ACE2 expression in the lungs than females (Devaux et al., 2020) (Figure 3).

**Figure 4:**
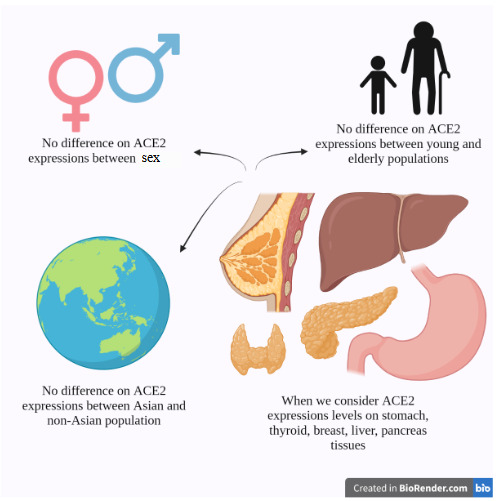
ACE2 expression discrepancies. Population, sex and age discrepancies in terms of ACE2 expressions in the thyroid, stomach, pancreas, breast and liver. With the aforementioned tissues considered, no difference was found between the sexes, between the young and elderly populations and between the Asian and non-Asian populations.

In TCGA datasets, ACE2 expression levels between the Asian and non-Asian races in five normal tissues (stomach, thyroid, breast, liver and pancreas) were also compared, and any significant difference between the races in any tissue was null (M. Y. Li et al., 2020). Likewise, in the TCGA datasets, it was observed that the ACE2 was not differentially expressed between males and females or between younger and older groups in any tissue (Figure 4) (M. Y. Li et al., 2020). However, the aforementioned study of Liu et al., (2010) has to be kept in mind when discussing the asexual tissue distribution of ACE2 (Liu et al., 2010). 

#### 2.3.5. The cardiovascular system and ACE2

Cardiac injury in viral infections is often related to the involvement of cardiomyocytes and systemic inflammations (Bojkova et al., 2020). The downregulation of ACE2 expression leads to diseases such as heart failure, hypertension, and diabetes (Bavishi et al., 2020; T. Yan et al., 2020). 

As for the studies regarding the cardiovascular system, single nucleotide polymorphism (SNP) research was applied to five different populations. Although the mentioned studies (Table 2) found no correlations, when another study that investigated patients who have Diabetes Mellitus type II (DM-II) was observed, it was found that there is an association between the rs2285666-G and rs2285666-A variants and septal thickness. The study also states that the G8790A polymorphism of the ACE2 gene plays a role in the pathogenesis of coronary artery disease in patients with DM-II (M.Y. Li et al., 2020). Although cardiogenic shock presents itself in low numbers, in patients with the SARS-CoV-2 infection, cardiac complications related to the underlying atherosclerotic cardiovascular diseases may significantly increase the severity of COVID-19 in vulnerable individuals (Bansal 2020; Hu et al., 2021). 

**Table 2:** Cardiovascular effects of ACE2 variants.

	Association with CVP*	No Association with CVP*
Females	rs1978124 (MI**)rs4646142 (MI**)	rs2285666 (MI**)
Males	rs4646156 (LVM-ST***)rs879922 (LVM-ST***)rs4240157 (LVM-ST***)rs233575 (LVM-ST***)rs1978124 (ACSAM****)rs2106809 (HCMP*****)rs6632667 (HCMP*****)	rs2285666 (LVM-ST***)

Note: The rs228566 allele has been highly associated with the risk of cardiovascular mortality in females, assuming the dose -dependent effect on the ACE2 protein (Vangjeli et al., 2011).In the analysis with regards to sex rs1978124 (1075A/G) and rs4646142 (16854G/C) were found to be risky for females, and it has been reported that females carrying the 1075AA and 16854GG genotypes are at higher risk in recessive models for myocardial infarction (Yang et al., 2006). Left ventricle mass and septal thickness of German males have a positive correlation with the rs4646156, rs4240157, and rs233575. The rs2106809 and rs6632667 are connected with hypertrophic cardiomyopathy in Chinese males. The rs2285666 A allele is connected with low interventricular septal thickness and low left ventricular mass (Vangjeli et al., 2011). The rs4240157, rs4646155 and rs48300542 variants could affect blood pressure and the rs2074192, rs233575 and rs2158083 variants are deemed pathological. The ACE I/D and ACE2 G8790A alleles are shown to increase blood pressure (Yongyue Chen & Yu, 2018; Pinheiro et al., 2019), which revealed the connections between the rs2106809 and the decreasing of blood pressure of patients who take ACE inhibitors. Female hypertensive patients who have the CC:CT heterozygous allele, have more blood pressure decrease than the TT homozygous individuals (Yongyue Chen & Yu, 2018). *: CVP: cardiovascular pathologies; **: MI: myocardial infarction; ***: LVM-ST: left ventricle mass-septal thickness; ****: ACSAM: acute coronary syndrome associated mortality; *****: HCMP: hypertrophic cardiomyopathy.

#### 2.3.6. ACE2, dna methylation and cytokines

Systemic lupus erythematosus (SLE) patients are seemingly more susceptible to SARS-CoV-2 as the gene expression in the T cells and in several tissues could be modified (Renauer et al., 2015). DNA hypomethylation and expressions of various genes may be higher in SLE patients than in the normal population. This is because the DNA methylase activity regulated by the ras- mitogen-activated protein kinase (MAPK) could be inhibited by MAPK inhibition (Deng et al., 2001). For instance, an abnormal inactivation for the normally inactivated X chromosome causes T cell autoimmunity due to increased CD40L expression in CD4(+) cells in women. As a starting point of such, research on the ACE2 gene suggests that female SLE patients have hypomethylation on the ACE2 promoter sites in their CD4(+) T cells (M. Zhao et al., 2014). Therefore, the ACE2 mRNA transcription is higher than normal. Especially in this situation, overexpression of ACE2 that functions as a receptor for SARS-CoV-2 may facilitate viral pathogenesis. Viral oxidative stress also can trigger hypomethylation and viremia (Y. Li et al., 2014). 

When the DNA methylation of the ACE2 loci in various tissues was compared with using the raw genome-wide DNA methylation array, DNA methylation was measured to be maximal in the lungs and minimal in the neurons and the leukocytes. However, it can be stated that the DNA methylation might be affected by tissue type, age, sex, and several other factors (Corley & Ndhlovu, 2020).

Another expected hypomethylation region in SLE are the cytokine genes. Hypomethylation of the IFN synthesis regulating genes and the nuclear factor kappa B (NF-κB) pathway genes that regulate cytokine release may cause an overtly strong immune response and cytokine storm (Renauer et al., 2015). Especially, the hypomethylation of cytokine genes in double-negative T cells that express interleukin 17 (IL-17), IL-18 and IFN gamma (IFN-γ), intensify the cytokine storm (Renauer et al., 2015).

## 3. Ace polymorphisms, in general

Polymorphism studies in countries that have a high COVID-19 prevalence showed that the insertion allele of the ACE deletion/insertion (D/I) polymorphism correlates with COVID-19 prevalence although the ACE enzyme has no direct impact on the SARS-CoV-2 pathogenesis. However, the ACE D allele shows negative correlation with COVID-19 prevalence. Controlled experimental studies have suggested that the ACE polymorphisms also affect the mortality rates in COVID-19 (Delanghe et al., 2020). The decrease of ACE2 expression and the deletion allele of ACE may cause the pulmonary disease to progress as an increase of ACE2, and an alteration in the ACE activity may be advantageous for the pulmonary disease progression (Gemmati et al., 2020). 

## 4. Type II transmembrane serine protease (tmprss2)

**Figure 5:**
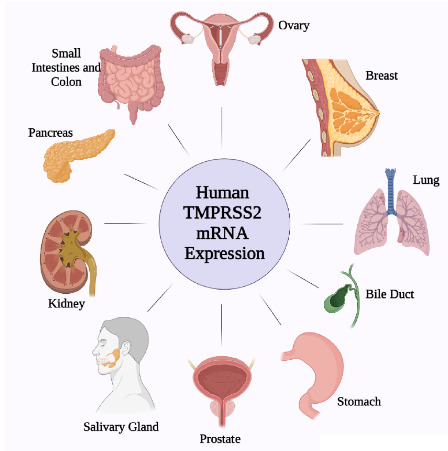
Human TMPRSS2 expression. Illustration showing the tissues where TMPRSS2 is expressed. The human TMPRSS2 mRNA is expressed in numerous tissues - including the prostate, breast, bile duct, kidney, colon, small intestine, pancreas, ovary, salivary gland, stomach, and lung- more often in epithelial cells.

Serine proteases of the trypsin-like family have been known as critical effectors of biological transactions as various as digestion, blood coagulation, fibrinolysis, and immunity (Antalis et al., 2011). Research to date has indicated that the androgen receptors are found in the respiratory epithelium, especially in type 2 pneumocytes and the bronchial epithelium, in humans and mice. However, the TMPRSS2 expression increased two-fold when lung cells were injected with androgen and TMPRSS2, known to contain an androgen-sensitive enhancer region, was also on the list of androgen target genes (Mikkonen et al., 2010) (Figure 5).

TMPRSS2, which is highly expressed on the luminal side of the prostate epithelium in cancerous cells, is found to be in rather high levels in cancerous prostate cells compared to non-cancerous cells (Lucas et al., 2008). TMPRSS2 is a member of the ETS family of oncogenic transcription factors, contributing to somatic gene rearrangements most commonly involving ERG with TMPRSS2, common to one of the most widely observed gene fusion events in solid tumors (Stopsack et al., 2020). Hormonally regulated TMPRSS2-ERG gene fusion prevalence is higher in European males which have primary prostate cancer (50%) in comparison with Asian or Black males. In males, prostate tumors which have TMPRSS2-ERG gene fusion also possess increased insulin/insulin dependent growth factor levels (Pettersson et al., 2013). 

TMPRSS2 also supports the virus’s entry into host cells by proteolytically dissociating and activating viral envelope glycoproteins. TMPRSS2 is essential for human coronaviruses - including SARS-CoV-2 - and influenza viruses to begin cell invasion and to reproduce.^3^NCBI (2021). Gene, TMPRSS2 transmembrane serine protease 2 [Homo sapiens (human)] [online]. https://www.ncbi.nlm.nih.gov/gene?Db=gene&Cmd=ShowDetailView&TermToSearch=7113. [accessed 11August 2020]. Hoffmann et al., (2020) reported that the emerging SARS-CoV-2 infection depends on the host cell factors such as ACE2 and TMPRSS2, and that TMPRSS2 exhibits a cumulative effect with furin in the course of virus entry. This furin activation facilitates entry of the virus into cell types that exhibit particularly low TMPRSS2 expression. Consequently, SARS-CoV-2 entry into the host cells is dependent on ACE2, which acts as a receptor for the viral S glycoprotein, and TMPRSS2, which provides a fusion of the host cell-viral cell envelopes (Hoffmann, Kleine-Weber, Schroeder, et al., 2020; Ou et al., 2020; J. Shang et al., 2020a; Walls et al., 2020; Zhou P. et al., 2020). There is a process utilized by SARS-CoV-2 to enter the host cell, which is defined as follows: the attachment to the cell using the (ACE2) receptor followed by creating a viral S protein using cellular TMPRSS2 (Hoffmann, Kleine-Weber, Schroeder, et al., 2020).

Immunohistochemical analyses showed that TMPRSS2 expression on lung bronchial epithelial cells is higher than that of the type II alveolar cells that produce the surfactant. Furthermore, there is no TMPRSS2 expression on the type I lung cells which are responsible for forming the lung surface (Bertram et al., 2012). Apart from lung and prostate cells, the protein of TMPRSS2 and TMPRSS2 mRNA were also found overall in the human body (Bertram et al., 2012; Lucas et al., 2008). These studies could explain the non-respiratory symptoms caused by the presence of coronaviruses and influenza viruses (Stopsack et al., 2020).

Stopsack et al., (2020) showed that the COVID-19 and influenza incidences are higher in males. In addition, it may be affected by the consumption of cigarettes and the high incidence in males might be explained by the fact that cigarette smokers are mostly male. TMPRSS2 expression is controlled by androgens and seems like it explains high male predominance. It is surprising that when the lung tissue is analyzed for the TMPRSS2 protein and its mRNA, there is no difference between both sexs (Stopsack et al., 2020). However, in post-menopausal females, the estrogen drop affected the TMPRSS2 expression, showing that the gene is also estrogen-respondent (Gemmati et al., 2020). 

**Figure 6:**
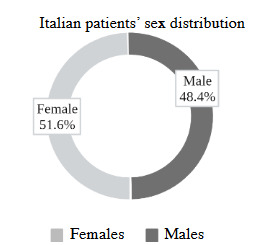
Epidemiological status in Italy (Patients). A pie chart displaying the sex distribution of patients in Italy. Constructed with the data from Istituto Superiore di Sanità of Italy.^1^ Containing discrepancies in comparison with the 9-month earlier study of Asselta et al., (2020) (Asselta et al., 2020). These distributions may help to assess both the sex-based and ethnicity-based differences in prognosis.^4^L’epidemiologia per la sanità pubblica, Istituto Superiore di Sanità (2021) Dati della Sorveglianza integrata COVID-19 in Italia [online]. Website: https://www.epicentro.iss.it/coronavirus/sars-cov-2-dashboard. [accessed 24 January 2021].

All around the world, poor prognosis has been associated with senility, comorbidities and the male sex. Also, Asselta et al., (2020) showed that Italy has a high male to female ratio of patients (male/female = 1.62) (Asselta et al., 2020) like other concurrent studies. When the same source was re-reviewed after 9 months (on 11 January 2021), it was seen that the ratio had changed (Figure 6). Asselta et al., (2020) claimed that the high male to female ratio was also similar to that of the 2003 SARS and 2012 MERS outbreak (Asselta et al., 2020). However, the website shows that the female patient numbers exceeding the number of male patients. This issue should be investigated in further detail in future studies, as there may be other underlying reasons for the change in rates. 

Nevertheless it has been said many times that males are more prone to having greater severity and mortality, independent of age (Jin et al., 2020). Such male predominance can also be corroborated with Baena et al., (2013)’s research about the relationship between TMPRSS2 expression and with androgenic and estrogenic stimulation, showing that TMPRSS2 expression is responsive to such stimulations (Baena et al., 2013). However, the current information is insufficient to reach a definitive conclusion about the effect of male to female ratios. When age is considered, Asselta et al., (2020) confirmed that there was no significant difference in the male to female ratio of TMPRSS2 levels among different ages (Asselta et al., 2020). According to the study of Schuler et al., (2021), the very low levels of TMPRSS2 expression in human infants suggest a mechanism by which neonates may be relatively protected against severe forms of COVID-19. 

In addition, females have stronger immune response to viral infections compared to males due to the more vigorous nature of their humoral and cellular immune responses (Klein & Flanagan, 2016). Compensating effects should not be overlooked in postmenopausal females as a result of low estrogen or in females receiving hormone replacement therapy (Asselta et al., 2020). 

Asselta et al., (2020), in addition to sex ratio, searched for TMPRSS2 gene variations between ethnicities (Table 3). They reported that the number of deleterious variants in Italians was significant lower compared to other Europeans, and that Italians may have higher TMPRSS2 protein/activity, which may contribute to a worse prognosis in the Italian population. Yet, the East Asian population has the lowest deleterious variants, so this decrease was even more evident. However, the number of the Italian population over 65 years of age is also twice as high as in the Hubei region, which may also be a factor explaining higher mortality rates as old age is a major risk factor for mortality (Asselta et al., 2020; Jernigan & Cox, 2015). 

**Table 3:** SNPs associated with the haplotypes from Asselta et al., (2020).

The SNPs which compose the “European Haplotype”	The SNPs that compose the “Other Haplotype”
rs463727, rs34624090, rs55964536, rs734056, rs4290734, rs34783969, rs11702475, rs35899679, rs35041537	rs2070788, rs9974589, rs7364083

Note: Table showing the core SNPs associated with the “European haplotype” and the “other haplotype” mentioned in Asselta et al., (2020). The “European haplotype”, which is not found in Asians and is thought to increase the TMPRSS2 gene expression in an androgen-spesific way and the “other haplotype”, associated with high TMPRSS2 levels. It has to be noted that rs2070788 is found to increase the risk both to human A (H7N9) and severe A(H1N1)pdm09 influenza (Z. Cheng et al., 2015). An interesting point is that the ratio of male patients to female patients in influenza A (H7N9) was found to be higher than 2 (Jernigan & Cox, 2015).

A quantitative method was developed by Ortiz-Fernández & Sawalha (2020) to cumulatively express genetic variations in ACE2 and TMPRSS2 expressions, and genetic differences among more than 2500 individuals from five different regions of the world were investigated. They explained that the East Asian populations had the maximum values for genetic descriptors of TMPRSS2 expression, while Africans showed a genetic predisposition for the lowest TMPRSS2 expression levels among populations and there was no difference between the male and female individuals in terms of TMPRSS2. These results suggest that a genetic component could help reduce reported cases of COVID-19 in Africa. However, non-genetic factors such as age and comorbidities may play a more important role than host genetic factors in determining the severity and outcome of disease in infected individuals (Ortiz-Fernández & Sawalha, 2020).

When frequently encountered exonic foci are examined, four SNP frequencies show a distinct difference in Italians and East Asians. Three out of four variants that show change are point mutations that do not ultimately cause a difference, while the other mutation is a missense mutation; p.Val160Met (Asselta et al., 2020). The p.Val160Met variant has recently been found to be associated with a mutation in TMPRSS2, particularly in terms of the risk of prostate cancer (FitzGerald et al., 2008) and the examination of this variant in patients may shorten the diagnosis time in high-risk individuals (Giri et al., 2011). In addition to this information, in two different cohort studies, they showed that high expressions of TMPRSS2-related single-nucleotide polymorphisms (SNPs) cause predisposition to the influenza virus (Z. Cheng et al., 2015).

Actually this transmembrane protein, although it plays a role in many pathways, does not have any essential duties in the cell (Kim et al., 2006). So, it can be inhibited without major complications in an effort to stop the spread of the virus. 

Hatesuer et al., (2013) studied TMPRSS2 in knock-out mice. They found that, if the TMPRSS2 was knocked-out in H1N1 infected mice, their infections were more modest and attenuated than that of the control group; also the mortality, loss of body mass and lung pathologies were found to be lesser (Hatesuer et al., 2013). As TMPRSS2 activates the influenza virus by cleaving hemagglutinin, this may suggest that the enzyme may contribute to virus invasion of human airways (Chaipan et al., 2009). 

## 5. The toll-like receptor (TLR) family

Toll-like receptors (TLRs) are a kind of pattern recognition receptors (PRRs) (Newton & Dixit, 2012). PRRs, which play a role in innate immunity, are activated by detecting pathogen-associated molecular patterns (PAMPs) and damage-associated molecular patterns (DAMPs), and oligomerizes multi-subunit complexes and creates signals that stimulate leukocytes. There are 10 TLRs (TLR1 - TLR10) defined in humans. Besides; TLR11, TLR12, and TLR13 defined in mice are included in the TLR family (Lim & Staudt, 2013). They generate different immune responses by stimulating signal pathways by detecting molecules such as teichoic acid (TA), arabinogalactan, lipopolysaccharide (LPS), unmethylated double-strand DNA (CpG), 23S rRNA, single-strand RNA (ssRNA), double-strand RNA (dsRNA) (Mahla et al., 2013). In our current problem, the COVID-19 pandemic, it is of great importance to investigate whether polymorphisms on TLRs effect the course of the disease.

**Figure 7:**
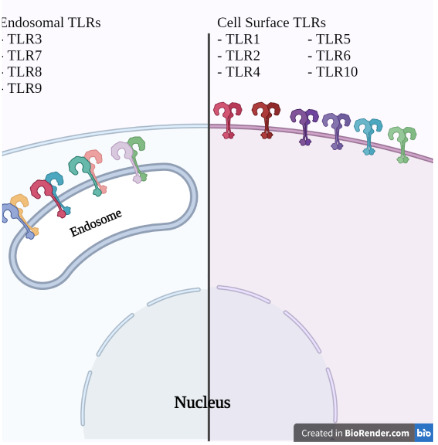
TLR types and localizations. Illustration showing the endosomal and cell surface toll-like receptor (TLR) types.

TLRs are expressed either on the cell membrane or the intracellular membrane of endosomes (Figure 7). Human endosomal TLRs (TLR 3, 7, 8, 9) mostly work to register the viral infections by identifying various nucleic acids from external sources (Fitzgerald & Kagan, 2020). The TLRs 7 and 8, which are endosomal receptors, detect viral nucleic acid PAMPs by detecting ssRNA. (Totura & Baric, 2012). TLR 8 can also detect RNAse T2 degradation products (Greulich et al., 2019). A meaningful insight into this will be that the Coronaviruses are viruses with single and positive stranded RNA ranging from 26-32 kb. (Perlman & Netland, 2009). The main reason that TLRs 7 and 8 are exceptions is that they are encoded in the X chromosome (C. H. Wang et al., 2011), and dosage compensation for X-linked gene products in women is achieved by random silencing of one of the two X chromosomes during the early development of female embryos therefore the fact that TLR 7 escapes the silencing of one of the X chromosomes in females makes this exception paramount (Souyris et al., 2018). This means that in females, TLR7 is expressed with respect to the genetic material of both of the X chromosomes. As this expression profile may cause sex-based differences in immune responses, causing females to have a more active response to single-stranded viruses (de Groot & Bontrop, 2020), these differences have to be thoroughly investigated. Another intriguing topic of interest regarding TLRs 7 and 8 is that they play an essential role in the pathogenesis in SLE, a disease more prominent in females, and they exhibit polymorphisms in the forms of copy number variations (CNV) and SNP (C. M. Wang et al., 2014). 

TLR3 recognizes double-stranded RNA (dsRNA) motifs from invading pathogens and enables activation of transcription factors. Although the role of TLR4 in pathogenic coronavirus infections is unclear, both receptors are sensitive in the pathogenesis of SARS-CoV and regulate pathogenesis. In addition, showed that mice lacking TLR3 and TLR4 had increased weight loss, mortality, decreased lung function, increased lung pathology, and higher viral titers (Totura et al., 2015). The SARS-CoV NSP16 protein shows 2-O’-methyltransferase activity and prevents the detection of cytosolic RNA by MDA5, thus escaping the immune system (Menachery et al., 2014). TIR domain-containing adapter molecule 2 (TICAM2) is a TLR adapter protein specific to TLR4. Increased susceptibility to SARS-CoV pathogenesis in TICAM2 deficient mice has been shown. The TLR4 signaling pathway plays a decisive role in the pathogenesis of viral infections. Variations in genes that affect this signal pathway can be determinative in disease progression (Gralinski et al., 2017). 

Structural changes on TLR2 and TLR4 due to polymorphisms in the TLR genes have an effect that can alter immune responses to some disease factors. Moreover, SNPs on these TLRs exhibit ethnic variations. Some Asian populations show high and low frequencies in comparison with Africans and Caucasians (Leung et al., 2005).

## 6. CD14 polymporphisms

CD14-159TT carriers have higher sCD14 levels than CD14-159CC carriers (Kabesch et al., 2004). The possible role of these polymorphisms is to change the inflammatory response in the individual. Although the mechanism of CD14 in response to viruses is not fully known, CD14-159CC polymorphism has been observed to show a higher frequency in patients with SARS-CoV compared to CD14-159TT. In line with these findings, it can be said that antiviral activity may be decreased in patients carrying CD14-159CC and may be characterized by a more severe SARS-CoV infection (Yuan et al., 2007).

## 7. AB0 blood groups

Previous studies showed that AB0 blood systems could affect the susceptibility of viral infections such as the Norovirus (Liao et al., 2020) and the Hepatitis B virus (Batool et al., 2017). Moreover, it was monitored that the A and D (Rhesus) blood groups have an effect on the severity of the West Nile virus prognosis (Kaidarova et al., 2016). It was also reported that the O blood group individuals are less infected by SARS-CoV virus (Y. Cheng et al., 2005). Therefore, Zhao et al., (2020) figured the AB0 blood groups could be a secondary biomarker for SARS-CoV-2 infection (J. Zhao et al., 2020). In addition, when the SARS-CoV-2 + individuals are compared to the general population, the A blood group had increased risk while the O blood group has shown lower risk than others (J. Zhao et al., 2020). Dai (2020) explained the susceptibility of SARS-CoV-2 and AB0 blood groups’ protective effect by implying that the O blood groups have an effect on ACE and ACE2 levels and indirectly changes IL-6 and C-Reactive Protein (CRP) levels; meanwhile, the A blood group has a significant effect on ICAM-1 and P-selectin levels (Dai, 2020). 

There are some conflicts about whether the AB0 blood group types affect the susceptibility to SARS-CoV-2 or the severity of the disease. Although there are studies that have reported that there is no correlation between susceptibility and the AB0 blood group types, some studies indicate that A and B blood group has a higher susceptibility for the virus. And even if the O blood type had protective effects on SARS-CoV, it was stated that people with the O blood group were at lower risk (Wu et al., 2020). Sardu et al., (2020) and Tanigawa et Rivas (2020) stated that blood type A has a significantly higher risk for COVID-19 compared to those who are not of blood type A, but that blood type O has a significantly lower risk for COVID-19 compared to those without blood type O. Although they have stated that AB0 blood group may have a higher sensitivity to COVID-19, more studies are needed (Sardu et al., 2020; Tanigawa & Rivas, 2020). Also, Zietz et al., (2020) found that the B blood group’s ratio was higher in COVID+ patients rather than COVID- individuals (Zietz et al., 2020). 

Likewise, there are also studies with conflicting results regarding the correlation between the severity of COVID-19 and the AB0 blood group types (Table 4). Some studies indicate that there is no significant relationship between the AB0 blood group types and the severity of the disease (Dai, 2020; Mendy et al., 2020; Wu et al., 2020). On the other hand, according to Zhao et al., (2020) the mortality is higher in the A blood group , again, blood type O has a lower risk and compared to AB0 blood types, AB blood type and B blood group are prone to high risk (J. Zhao et al., 2020). Further investigations are necessary to shed light on such evidence.

**Table 4:** AB0 polymorphisms and ACE inhibitors.

AB0 blood group polymorphisms	rs495828, gene promoterrs8176746, exon 7

Note: Select AB0 blood group polymorphisms that affect the treatment response of ACE inhibitors (Gemmati et al., 2020).

## 8. Individual Discrepancies Related to Lifestyles and Origins 

### 8.1. Smoking

The fashion in which smoking effects COVID-19 and the aftermath is still unknown. Early data have not ensured enough proof on the connection between smoking and the detrimental effects of COVID-19 (Lippi & Henry, 2020; Vardavas & Nikitara, 2020). Comprehending these factors is crucial for analyzing the clinical risk, evolving the information about clear public health, and finding targets for intervention. Prior and present smoking raise the risk of viral (Abadom et al., 2016; Denholm et al., 2010) and bacterial (Feldman & Anderson, 2013) respiratory infections and they are associated with worse outcomes for the ones who are infected. The fact that smoking decreases respiratory immune protection along with the behavioral attitudes of smoking may be a reason for intensified transmission (Beamer et al., 2015).

It has been reported that smokers are 1.5 times more likely to develop severe respiratory disease from COVID-19 infection than non-smokers, and are approximately 2.5 times more likely to be hospitalized in intensive care units (Vardavas & Nikitara, 2020). However, this has not been persistently reported (Lippi & Henry, 2020). A review stated that the signs about disease severity in patients who are hospitalized for COVID-19 is higher in prior/present smokers than those who had never consumed tobacco before; nevertheless, there was no sufficient evidence to come to a decision on infection, hospitalization or mortality (Simons et al., 2020). A meta-analysis indicated that smoking is a risk factor for the progression of COVID-19 disease due to its adverse effects on pulmonary immune function, and that smokers have an approximately 2 times higher probability of COVID-19 progression than non-smokers (Patanavanich & Glantz, 2020).

When analyzed by self-report in a population sample, present smoking was separately linked with a higher probability of COVID-19 infection. Chances of contracting COVID-19 were 3.5 times greater in present smokers without post-16 qualifications than those who had never smoked before after modification for covariates, but they did not differ fundamentally by smoking status in patients with post-16 qualifications. Socio-economic inequalities were clearly found in those without post-16 qualifications. Smokers stated lesser obedience to principles although being more concerned than non-smokers about getting infected and becoming critically ill due to COVID-19. COVID-19 was also associated with gradual increase in smoking frequency as a result of stress, especially with the ones without post-16 qualifications (Jackson et al., 2020).

The population-based studies of Gaiha et al., (2020) ensured that young users of e-cigarettes during youth and binary users of e-cigarette and cigarettes are at higher risk of COVID-19. Surprisingly, exclusive ever-use of combustible cigarettes was found to be only correlated with COVID-19 related testing, considering that former 30-day use, ever-use of e-cigarettes and binary use were correlated with COVID-19 testing and positive diagnosis (Gaiha et al., 2020). There are some possible reasons why both binary use and e-cigarette use were correlated with getting infected with COVID-19: amplified exposure to nicotine and alternative chemicals in e-cigarettes unfavorably influences lung infection and it is clear that e-cigarettes are just as harmful as tobacco cigarettes (Reinikovaite et al., 2018), COVID-19 outspreads as a consequence of recurrently touching of one’s hands to the mouth and face (Berlin et al., 2020). Sharing equipment is again a common implementation by the whole of youth e-cigarette users (McKelvey & Halpern-Felsher, 2020).

In a recent study, there is clear controversy that a population in the UK revealed smoking to be unquestionably linked with higher COVID-19 mortality, falling under age and sex; yet at the same time linking smoking with a lessened risk for COVID-19 mortality (Williamson et al., 2020). The defensive connection maintained stable after a few separate adjustments to the model. While smoking has several plausible biological mechanisms and defense effects associated with the less severe disease of COVID-19, it is improbable to weigh down the proven adverse effects of smoking. Obviously, evidence about this topic is restricted and thus confusing; more studies should be done and it should not be concluded that smoking has a protective effect (Usman et al., 2020).

### 8.2. Obesity and diabetes

As factors such as blood groups, age, race, sex, and smoking have an effect on COVID-19, body mass index (BMI) may also be an important factor for COVID-19. Early research revealed that obesity (having a BMI higher than 30) may be a risk factor for patients younger than 60 years old (Lighter et al., 2020). A cohort study of 489,796 adults from UK Biobank attempted to reveal the relationship between body mass index categories and central obesity with COVID-19. According to the results of the study, adults with high BMI had a greater dose-response increase in the risk of severe COVID-19 than those with a normal weight (Zhu et al., 2020).

Obese patients at the time of their hospitalization had higher plasma CRP levels and lower lymphocyte counts, which are considered as the two early indicators of severe COVID-19. It has also been shown that patients with obesity have a greater duration of hospital stay and a more severe disease course (Gao et al., 2020). In addition to these effects, it has been stated that fatty liver disease caused by obesity increases the severity risk for COVID-19 six-fold. Moreover; the diet associated with obesity, which consists mainly of fats, activates the innate immunity while disrupting the adaptive immunity, which, in turn, causes chronic inflammation and the vulnerability of the host’s defense (Engin et al., 2020).

Diabetes patients are also among the groups with high risk in the COVID-19 pandemic associated with obesity (Acharya et al., 2020). COVID-19 patients without other comorbidities but with diabetes have been shown to be at risk of more severe pneumonia, release of tissue-damage-related enzymes, excessive uncontrolled inflammatory responses, and hypercoagulation due to dysregulation of glucose metabolism. Serum levels of biomarkers such as IL-6, CRP, D-dimer, and serum ferritin coagulation index were significantly higher than those without diabetes. This has shown that diabetes patients are more susceptible to cytokine storm in COVID-19 infections (Guo et al., 2020).

### 8.3. Ethnicity

The hosts’ genetics may also take part in the disease displayed as ethnicity. However, considering that ethnicity may also be treated as a social topic in the modern healthcare systems, looking at ethnicity from only a genetic standpoint would cutback the observable effects it has on the outcome of this disease. 

From the genetics view point alone, the first thing that can be noted is that there is a Neanderthal-derived risk haplotype arising from chromosome 3, which presents itself at a frequency of 30% in South Asia, 8% in Europe, 4% in mixed Americans. However, although this rate rises to 63% in Bangladesh, the rate in Africa is almost zero (Zeberg & Pääbo, 2020). Furthermore, Amodio et al., (2021) deduce that the Neanderthal haplotype may increase the severity of the disease. In order to confirm, the risk of dying from the disease was compared in the Bangladeshi population residing in the UK with the general population (Amodio et al., 2021). The reason that this haplotype is deemed as a “risk haplotype” is the fact that the 3p21.31 gene cluster was identified as a genetic susceptibility locus for patients with COVID-19 that have respiratory failure by the “The Severe Covid-19 GWAS Group” (2020) (“Genomewide Association Study of Severe Covid-19 with Respiratory Failure,” 2020). Mortazavi et al., (2021) also stated that the genetic disposition caused by the Neanderthal heritage in the Iranian population may play an increasing role in the course and the severity of the disease in Iran (Mortazavi et al., 2021). However, Zeberg and Pääbo (2021) concluded that a Neanderthal haplotype on chromosome 12 containing the genes OAS1, OAS2 and OAS3 play a protective role against the disease (Zeberg & Pääbo, 2021). These haplotypes may contribute to the individual differences in both the intra- (as individuals in the same population may have different ancestries) and inter-population level.

The fact that there is a non-uniform effect between Black and Asian populations and that these ethnicities are disproportionately more severely affected by this disease has been clarified by several studies (Abedi et al., 2020; Lassale et al., 2020; Raisi-Estabragh et al., 2020). However, one study claimed that once hospitalized, the Black population mortality rates were among the “norm”, and there is minimal racial difference in the severity of comorbidity or disease (Silver et al., 2020). This difference shows how the socioeconomic status of the patients affect the outcome of COVID-19. Both Lassale et al., (2020) and Raisi-Estabragh et al., (2020) showed that lower socioeconomic conditions experienced in minority ethnic groups in the UK (i.e. Black, Asian and Other populations) are associated with worse global health problem (Lassale et al., 2020; Raisi-Estabragh et al., 2020). In addition and as an example for how socioeconomic status can further affect the disease is made visible by periodontal disease, it is associated with lower socioeconomic status (Almerich-Silla et al., 2017) and it is thought to be associated with COVID-19 infections (Kara et al., 2020). 

However, the ethnicity based differences cannot be adequately explained by socioeconomic factors only (Raisi-Estabragh et al., 2020), and the associations were attenuated when the socioeconomic factors were considered (Lassale et al., 2020). Thus, there is a need for this topic to be thoroughly explored. These discoveries highlight the importance of acknowledging all the patient traits including their ethnicities and socioeconomic status as they might show the need for extra preventive measures to be taken. 

## 9. Conclusion and future prospects 

The epidemiological factors –e.g., age, sex, race, the polymorphisms, the blood types, and individual differences have all been in discussions about affecting the progression and the course of COVID-19 both individually and collectively, as their effects are mostly interwoven. Likewise, it cannot be denied that the social parameters impose sanctions on the disease too. 

Personalized medicine is in the form of appropriate medication at the right time, for the right patient, according to the personal diagnosis. The medical history of each patient and the prognosis of the disease are not one and the treatment approach should be arranged according to patient-specific parameters. In this pandemic we are in, a consensus on the appropriate drug and appropriate treatment approach for the SARS-CoV-2 infection has yet to be reached. While determining the appropriate treatment approach, parameters varying according to individuals should be the primary focus and the correct drug and treatment should be administered accordingly. 

To achieve this ideal of personalized medicine, some basic tests for determining the variants of the patients can be done and a thorough anamnesis should be recorded. Special drug regimens regarding the patient’s status and characteristics can, thus, be given. Further research is needed to further extend our knowledge about this disease and to enhance the medical amenities that can be provided to patients.

## Funding statement

The authors declare no specific funding for this work.

## Authors’ contribution

All authors contributed to the study conception and design while Pelin KILIC additionally conducted the overall supervision of the review. Material preparation, data collection and analysis were performed by Doruk ALTIOK, Elif Zeynep SAVCI, Büşra ÖZKARA, Kamil ALKAN, Dilara Sultan NAMDAR, Gizem TUNCER, Buğrahan Regaip KILINÇ, Evren SUİÇMEZ, Güneysu ÇETİN, Sinan ÜNAL, Beyza DONMUŞ, Zeynep Yağmur KARAGÜLLEOĞLU, Dilruba Beyza UNCUOĞLU, Cansu TEKELİ, Ayşegül Hanife MENDİ, Vahdi Umut BENGİ, Güldane CENGİZ SEVAL, Pelin KILIÇ, Evrim GÜNEŞ ALTUNTAŞ, Devrim DEMİR-DORA and all authors commented on previous versions of the manuscript. All authors read and approved the final manuscript. 
